# Application of the S-N Curve Mean Stress Correction Model in Terms of Fatigue Life Estimation for Random Torsional Loading for Selected Aluminum Alloys

**DOI:** 10.3390/ma13132985

**Published:** 2020-07-04

**Authors:** Michał Böhm, Krzysztof Kluger, Sławomir Pochwała, Mariusz Kupina

**Affiliations:** 1 Department of Mechanics and Machine Design, Faculty of Mechanical Engineering, Opole University of Technology, 45-271 Opole, Poland; k.kluger@po.edu.pl; 2 Department of Thermal Engineering and Industrial Equipment, Faculty of Mechanical Engineering, Opole University of Technology, 45-271 Opole, Poland; s.pochwala@po.edu.pl; 3 Department of Mechanics and Structural Engineering, Faculty of Civil Engineering and Architecture, Opole University of Technology, 45-061 Opole, Poland; m.kupina@po.edu.pl

**Keywords:** fatigue of materials, AW-6082-T6, AW-2017A-T4, AW-2024-T3, mean stress, aluminum alloys, S-N curve

## Abstract

The paper presents the experimental fatigue test results for cyclic constant amplitude loading conditions for the case of the torsion of the PA4 (AW-6082-T6), PA6 (AW-2017A-T4) and PA7 (AW-2024-T3) aluminum alloy for a drilled diabolo type test specimen. The tests have been performed for the stress asymmetry ratios *R* = −1, *R* = −0.7, *R* = −0.5 and *R* = −0.3. The experimental results have been used in the process of a fatigue life estimation performed for a random generated narrowband stress signal with a zero and a non-zero global mean stress value. The calculations have been performed within the time domain with the use of the rainflow cycle counting method and the Palmgren−Miner damage hypothesis. The mean stress compensation has been performed with the S-N curve mean stress model proposed by Niesłony and Böhm. The model has been modified in terms of torsional loading conditions. In order to obtain an appropriate *R* = 0 ratio S-N curve fatigue strength amplitude, the Smith−Watson−Topper model was used and compared with literature fatigue strength amplitudes. The presented solution extends the use of the correction model in terms of the torsional loading condition in order to obtain new S-N curves for other *R* values on the basis of the *R* = −1 results. The work includes the computational results for new fatigue curves with and without the mean stress effect correction. The results of the computations show that the mean stress effect plays a major role in the fatigue life assessment of the tested aluminum alloys and that the method can be used to assess the fatigue life under random conditions.

## 1. Introduction

The central motivation of this paper is the fatigue life assessment of the PA4, PA6 and PA7 aluminum alloys under random loading conditions under torsion stress with a global non-zero mean stress value. Aluminum alloys have many applications these days due to their weight, and they can be used in the automotive, maritime, as well as in the aviation industry. These applications are very often connected with the bearing of torsional forces, as the materials are being subjected to variable loading conditions. These conditions cause the premature failure of the material, which can be explained as the effect of material fatigue. This can be visualized as we analyze the material for the initiation of micro-cracks, which propagate into larger cracks which later on cause the failure of the material. As correctly stated by Wang et al. [1]: “failure analysis and fatigue life prediction are necessary and critical for engineering structural materials”. That is why we want to omit a situation where the initiation of a crack is premature and that is why we should always perform a fatigue assessment procedure in terms of the initiation process due to certain loading conditions. Many fatigue assessment techniques have been developed during the last century and up to now. Zhang et al. have presented a strain-based model to account for the non-zero mean strain effect in variable loading [2]. Xing et al. presented a new method to predict bending fatigue with spatial distributions [3]. Zhang et al. presented a novel damage accumulation model that takes into account amplitudes below the fatigue limit with the use of the modified Chaboche model [4]. Benasciutti et al. have presented a new frequency analysis of random loading models in practice [5]. New methods for fatigue life prediction without material constants have been the domain of the group of Yu et al. [6]. Methods based on the fatigue mechanism for multilayered materials have been presented by Li et al. [7]. Real-time online calculation methods for variable loading such as the one presented by Xue et al. [8] are getting more interest. This can also be noticed for methods combining low and high cycle regimes, such as the one presented by Zhu et al. [9]. Most of these methods are based either in the time or frequency domain. They use the cycle counting method (time domain) [10] or power spectral density (frequency domain) [11] to calculate the damage degree. Beside this, these methods use sophisticated calculation algorithms, which also require material information obtained during experiments [12,13]. These are usually performed for standardized specimens tested under constant amplitude loading conditions. As we go through the literature, we can find a number of fatigue experiments performed for torsion or similar conditions for the materials that are being analyzed in this paper. Rozumek and Faszynka [14] have tested the AW-2017A-T4 in terms of surface crack growth, taking into account the mean stress effect. Kluger has performed tests for the multiaxial state of bending-torsion for the AW-2017A-T4 and AW-6082-T6 [15], also with the mean stress effect. Szusta and Seweryn have tested the AW-2024-T3 in terms of low cycle behavior under axial-torsional fatigue loading [16]. The same aluminum alloy was also a point of interest in low cycle fatigue research by Pec et al. [17], where they analyzed it in terms of various loads, also in the multiaxial state of tension-compression. In some cases, it is also possible to test these specimens under preregistered loading histories obtained from strain gauges which can be glued to the material before its exposure to real loading conditions. The last case is usually very difficult to obtain if we are dealing with a new material or a new construction in which we do not know the distribution of the real-time loading conditions. This is the moment in which we have to generate a random stress time signal or a power spectral density dependent on the calculation domain we are going to choose. Many people argue about the fact that the time domain calculation methods are more precise when compared to the frequency domain, as we can read in the papers by, e.g., Gadolina et al. [18]. In both cases, we can assess the fatigue lifetime with the use of a random signal, but in the case of the time methods we need the time history, which is not a requirement for the frequency domain methods. If we want to create an S-N curve for the random loading signal, in the case of the frequency domain we are operating on a rescaled power spectral density and in the case of the time domain on a rescaled random stress history. Nevertheless, factors such as the mean stress or overloading influence can be currently compensated in a simpler way with the use of the time domain, and this is this method’s strength [19]. Beside this, the weakness of the time domain lies in the fact that it is more time consuming when compared with the frequency domain [18]. In the course of the paper, the authors are going to introduce the well-known rainflow cycle counting method and its application in the process of damage degree calculation with the use of the Palmgren−Miner damage hypothesis in the time domain. The experimental results of the fatigue tests are being used in order to obtain fatigue coefficients which will allow for the fatigue life assessment, with the use of the Niesłony-Böhm model, of two S-N curves for the narrowband-generated signal for two cases of zero mean global stress value, as well as for the second case, in which the mean stress value is equal to the highest stress amplitude value for all three materials [20].

## 2. Materials

It is well known that aluminum alloys do not have a visible fatigue limit, as is usually the case for steel materials. While analyzing these alloys, we can take an arbitrary value of two million cycles as the fatigue limit as presented, e.g., by Boller and Seegers [21] in their reports. Nevertheless, in the forthcoming descriptions in this chapter, the fatigue strengths (for aluminum alloys, we are going to use this description instead of the limit) are being obtained on the basis of experimental results as the highest obtained cycle value with the corresponding stress amplitude. These values have been obtained during cyclic fatigue tests performed on an experimental test stand patented by the Opole University of Technology. The description of aluminum alloys used in this research, PA4 (AW-6082-T6, Kęty Group, Kęty, Poland), PA6 (AW-2017A-T4, Kęty Group, Kęty, Poland) and PA7 (AW-2024-T3, Kęty Group, Kęty, Poland), under different international standards is presented in Table 1. Important mechanical properties are presented in Table 2. These properties have been determined either by our own experiments for PA4 and PA6 and compared with the material suppliers’ certificate or, as was the case for PA7, we gave the suppliers certificate values. The chemical composition of the tested alloys is presented in Table 3. It is given on the basis of the suppliers’ certificate.

## 3. Methods

The planned torsional stress state which should be obtained during the experimental tests was connected with the expected stress asymmetry ratio *R*, which can be defined via the use of the maximum *τ_max_* and minimum *τ_min_* value of the stress during the cyclic tests:

(1) $$R=\frac{\tau_{min}}{\tau_{max}}$$

The ratio can also be connected with the value of the mean stress *τ_m_* and stress amplitude *τ_a_*:

(2) $$R=\frac{\tau_{m}-\tau_{a}}{\tau_{m}+\tau_{a}}$$

As for the mean stress and stress amplitude, they can be defined as:

(3) $$\tau_{m}=\frac{\tau_{min}+\tau_{max}}{2}$$

(4) $$\tau_{a}=\frac{\tau_{max}-\tau_{min}}{2}$$

That means that in order to obtain a certain ratio, we need to often add an extra static stress value to the material. The torsional stress value is obtained during experimental tests under a controlled torsional moment, and therefore it can be calculated with the use of Equation (5):

(5) $$\tau_{a}=\frac{M_{t}}{W_{o}}$$

where *M_t_* is the torsional moment, and *W_o_* is the section modulus for the torsion calculated with the use of the outer diameter *D* and inner diameter *d*:

(6) $$w_{o}=\frac{\pi \cdot \left(D^{4}-d^{4}\right)}{16D}$$

In order to take the mean stress effect into account, we need to transform the stress amplitude. The literature presents a number of transformation models which are widely used. These models use mechanical properties of materials such as the yield strength or ultimate strength, as in, for instance, the Goodman [22], Gerber [23] or Soderberg [24] models. There are a number of models that take into account the effect of fatigue properties, such as the Morrow [25] model, which takes the value of the fatigue stress coefficient. Some mean stress compensation models can also be defined for the strain energy, like the well-known Zhu et al. model [26]. In this paper, we are going to use the Niesłony−Böhm [20] transformation model in terms of torsional stress, which takes into account the values of the fatigue strength amplitude for *R* = −1 (*τ_af_*), as well as any other fatigue strength amplitude. The model works on the basis of the interpolation of the values between two S-N curves. In the case where we compensate for the *R* values between −1 and 0, we will obtain an interpolated value for every compensated amplitude. In the case of this study, it will be for *R* = −1 (*τ_af_*) and *R* = 0 (*τ_afR_* = 0). It can be presented in the iterated form of Equation (7):

(7) $$\tau_{aTi}=\left[1+\tau_{m}\cdot \left(\frac{1-R}{R+1}\right)\cdot \frac{\tau_{af}-\tau_{afR=0}}{{\left(\tau_{afR=0}\right)}^{2}}\right]\cdot \tau_{ai}$$

The values in the brackets are constant and do not change with every loading cycle. Generally, we can obtain the fatigue strength values from different sources, e.g., Boller and Seeger [21] material table data or websites which publish experimental fatigue test results, such as the Fatlim selector explained by Tomcala et al. [27]. In the case of *R* = 0, the model is simplified into the form:

(8) $$\tau_{aTi}=\left[1+\tau_{m}\cdot \frac{\tau_{af}-\tau_{afR=0}}{{\left(\tau_{afR=0}\right)}^{2}}\right]\cdot \tau_{ai}$$

In order to obtain the missing value of the fatigue strength amplitude for *R* = 0, we have taken two approaches, the first being with the Smith−Watson−Topper model (SWT). This model is mainly suggested for axial loading cases and is rarely discussed or used for the pure torsional state, but we can find some recent papers, such as Gates and Fatemi [28] or Wu et al. [29], which discuss its use in terms of pure torsional load mean stress compensation. The second approach consisted in reading the missing cyclic fatigue strength data for our tested materials from the book by Niezgodziński [30]. Thus, in order to only obtain the missing fatigue strength for the Niesłony−Böhm model using the first approach, we can calculate it with the use of Equation (9) [31]:

(9) $$\tau_{aT}=\tau_{af}=\sqrt{\left(\tau_{a}+\tau_{m}\right)\cdot \tau_{a}}$$

Due to the fact that in the case of *R* = 0, *τ_a_* = *τ_m_* = *τ_afR_* = 0, the model takes the form of Equation (10):

(10) $$\tau_{aT}=\tau_{af}=\sqrt{\left(\tau_{afR=0}+\tau_{afR=0}\right)\cdot \tau_{afR=0}}$$

which leads to:

(11) $$\tau_{afR=0}=\frac{\tau_{af}}{\sqrt{2}}$$

**Figure 1:**
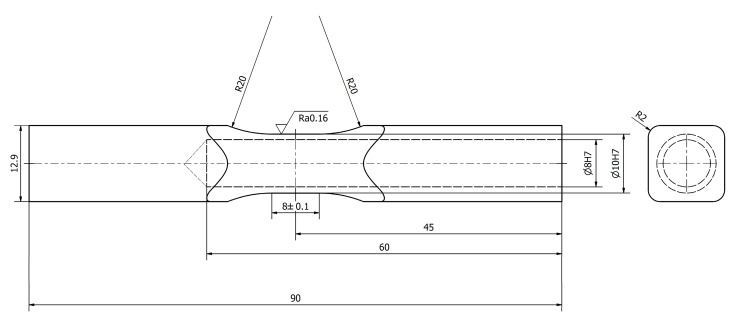
Drilled fatigue diabolo type test specimen (dimensions in mm).

**Figure 2:**
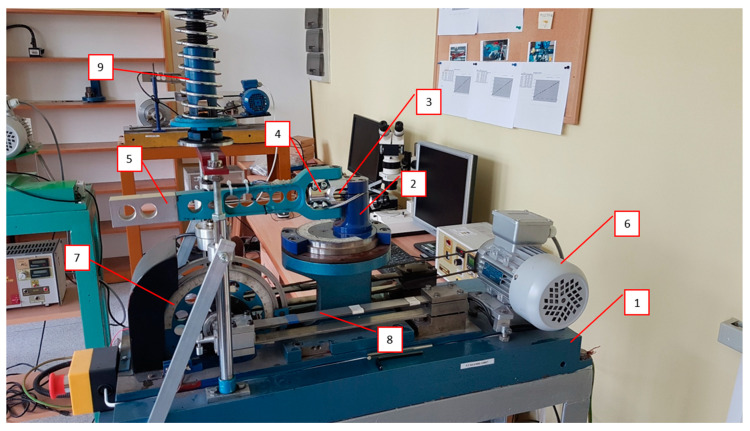
The fatigue test stand MZGS-100 for the bending and torsional tests, where: 1—bed, 2—rotational head with a holder, 3—specimen, 4—holder, 5—lever (effective length = 0.2 m), 6—motor, 7—rotating disk with mounted unbalanced mass, 8—flat springs, 9—spring actuator.

**Figure 3:**
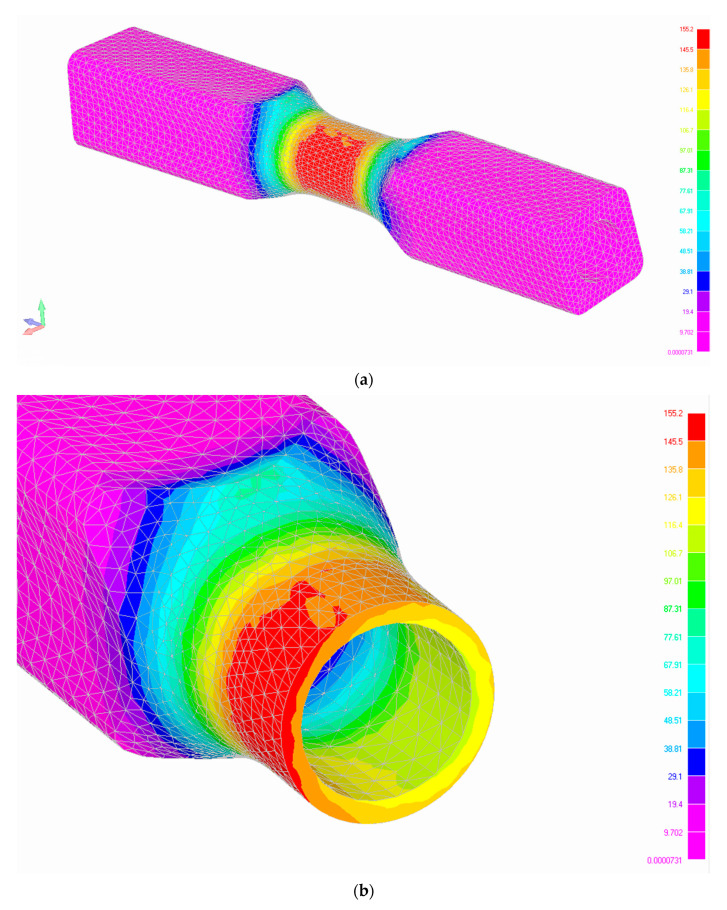
(**a**) Shear stress distribution (MPa) along the specimen for the maximum possible torsional moment (**b**) and the cross-section view of the sample at the spot with the maximum value.

For the torsional fatigue tests, we have used a modified diabolo specimen. The shape and dimension of the new type of experimental diabolo specimen is presented in Figure 1. We can find a variety of similar samples presented also inter alia in Fojtik et al. [32]. The sample has been drilled in order to obtain a maximum shear stress concentration in the cross section area during the torsional tests on the test stand MZGS-100, which is presented in Figure 2. The test stand allows us to perform cyclic bending or torsional tests as well as multiaxial cyclic tests with different values of phase shifts between the axes. The tests are controlled with a constant value of the bending (*M_b_*) or torsional moment (*M_t_*). The maximum allowable value of the bending moment is 80 Nm, and the value of the static moment is 60 Nm. The maximum frequency that can be obtained during the bending or torsional test for the stand is 29 Hz. Taking the limitations of the stand into account, a simple static finite element method (FEM) calculation has been performed in order to assess what maximum stress values can be obtained during the experimental tests for the dimensions and mechanical properties of the specimen. The shear stress distribution along the specimen is presented in Figure 3a. The view of the cross section in the middle of the specimen is presented in Figure 3b. The computations have been performed with the use of the Siemens FEMAP v.11 program and the NX NASTRAN solver. The force that was simulating the leverage work (84.5 N) was simulated with the use of an independent node placed at a distance of 200 mm to the specimen position’s center in the holder, at the point of the lever where it connected to the rotating disks, and this node was connected to the surface of the specimen with rigid elements. As one can note, our test stand imposes the torsion with the use of a lever, and due to this a parasitic shear stress remains present, which results from bending caused by the lever. The influence is negligible, but as one can notice this is the cause for such a distribution.

The tests were performed under cyclic loading conditions for a stress asymmetry ratio value of *R* = −1 and for individual results for *R* = −0.7, *R* = −0.5, *R* = −0.4 and *R* = −0.3. These results were used in order to obtain an S-N curve for *R* = −1, with its values of the slope *m*, fatigue strength stress amplitude, as well as the fatigue strength cycle value. This information has been used in order to calculate the fatigue life with the use of the rainflow cycle counting method and the Palmgren−Miner damage accumulation hypothesis. The rainflow cycle counting method was first introduced by Endo and Matsuishi [33]. Since then, it has been refined and is now one of the primary cycle counting methods due to the fact that it takes into account cycles as well as half cycles [34]. The Palmgren−Miner hypothesis [35] is one of the most popular damage accumulation hypotheses due to its simplicity and reliability, as it is independent of the loading sequence. It takes into account the slope value *m*, the fatigue strength stress value *τ_af_*, the fatigue strength *N_f_*, as well as the transformed stress amplitude resulting from the mean stress effect *τ_aiT_*:

(12) $$D_{P-M}(N_{0})=\{\begin{array}{c} \sum_{i=1}^{j}\frac{n_{i}}{N_{f}{\left(\frac{\tau_{af}}{\tau_{aiT}}\right)}^{m}}\;\;for\;\tau_{aiT}\geq a\cdot \tau_{af}\\ 0\;\;\;\;\;\;\;\;for\;\tau_{aiT}<a\cdot \tau_{af} \end{array}$$

The final formula for the fatigue life assessment takes the following form:

(13) $$N_{cal}=\frac{N_{obs}}{D_{P-M}\left(N_{0}\right)}$$

where *N_obs_* is the observation time taken from the rainflow algorithm.

**Figure 4:**
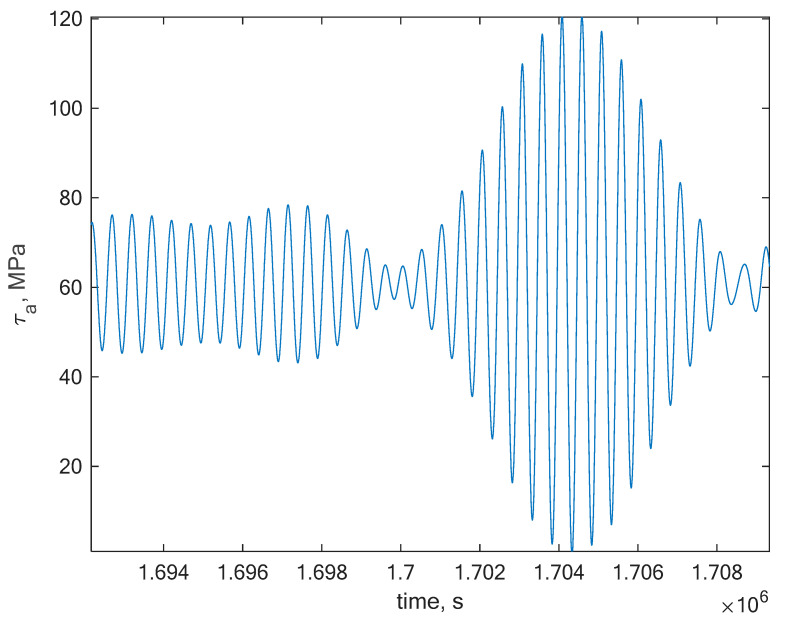
Section of the generated narrowband stress signal with a dominating frequency of 5 Hz generated in order to calculate the lifetime via the rainflow method for *R* = 0.

To perform the main task of the paper, which is to assess the fatigue life for a random signal, a narrow band stress signal was generated. The dominating frequency of the signal was 5 Hz. A section of the generated signal is presented in Figure 4. The signal has been rescaled each time due to a maximum value of the stress amplitude, which was expected. Thanks to this, it was possible to create an S-N curve, which allowed us to outline a safe area for the generated stress signal values which could be applied to the material beneath the curves [36].

## 4. Results

**Figure 5:**
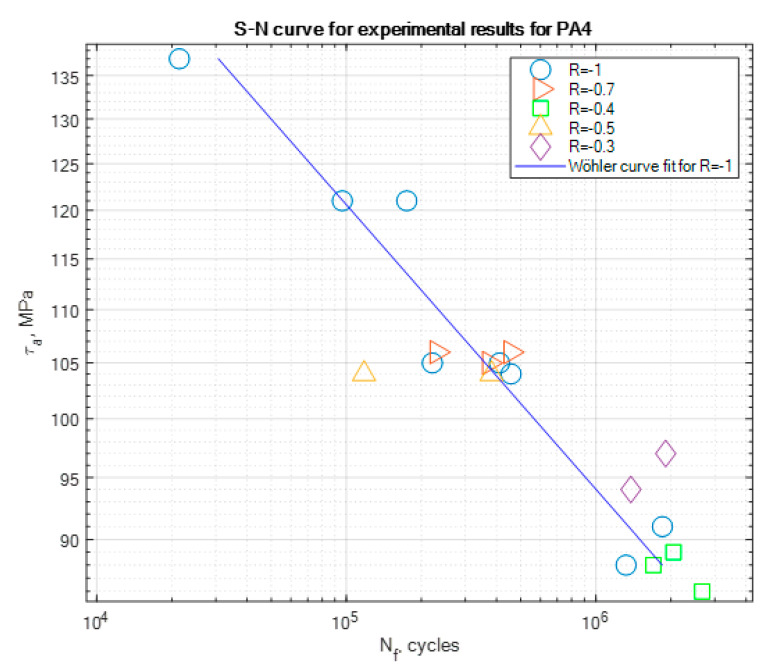
S-N curve for the experimental results for PA4 for *R* = −1, with the marked results for other tested stress asymmetry ratios.

**Figure 6:**
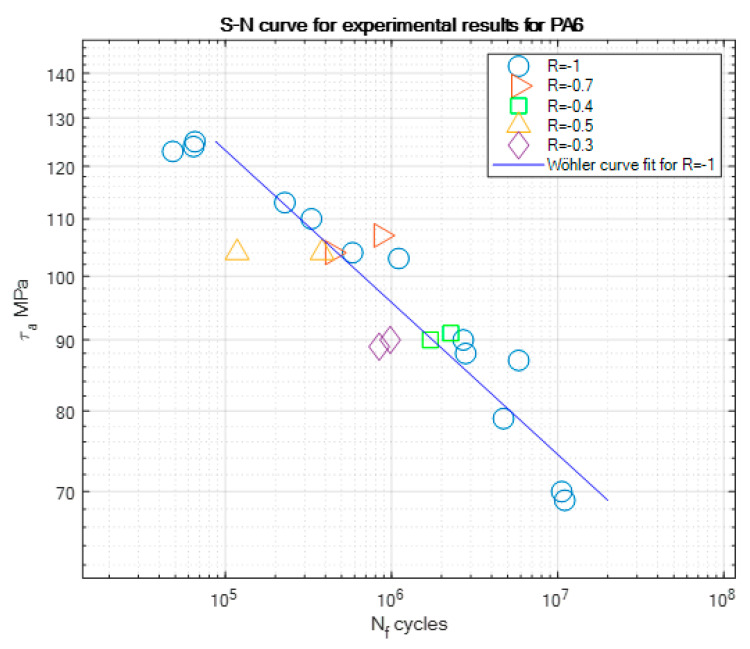
S-N curve for the experimental results for PA6 for *R* = −1, with the marked results for other tested stress asymmetry ratios.

**Figure 7:**
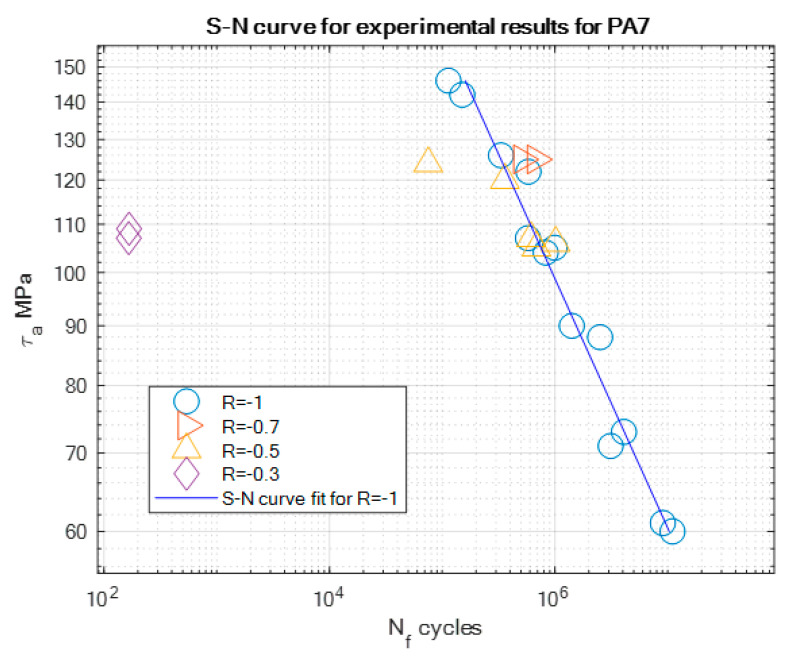
S-N curve for the experimental results for PA7 for *R* = −1, with the marked results for other tested stress asymmetry ratios.

The experimental results of the fatigue tests are presented in Table 4, Table 5 and Table 6 and graphically in the form of the S-N curves in Figure 5, Figure 6 and Figure 7. These curves are presented in a semi-logarithmic form. They present the experimental fatigue curve for a given aluminum alloy for the asymmetry ratio *R* = −1 and also singular points of experiments performed under different asymmetry ratios. At least two samples have been tested for each asymmetry ratio level and serve as illustrative values beside the S-N curve obtained for *R* = −1. Some outliers, e.g., the two points at *R* = 0.3 in Figure 7, have not been the point of our interest, but we are planning further tests for PA7 under this loading ratio in the future.

On the basis of the experimental results, S-N curve coefficients such as the slope *m* and fatigue strength stress amplitude *τ_af_* corresponding to the value of the fatigue strength *N*_f_ have been calculated and are presented for all the tested materials in Table 7. We can note that the slope values of PA4 and PA6 are close to each other, which can be explained by the fact that they have similar mechanical properties, such as the ultimate strength *R_m_* or yield strength *R_e_*. 

**Figure 8:**
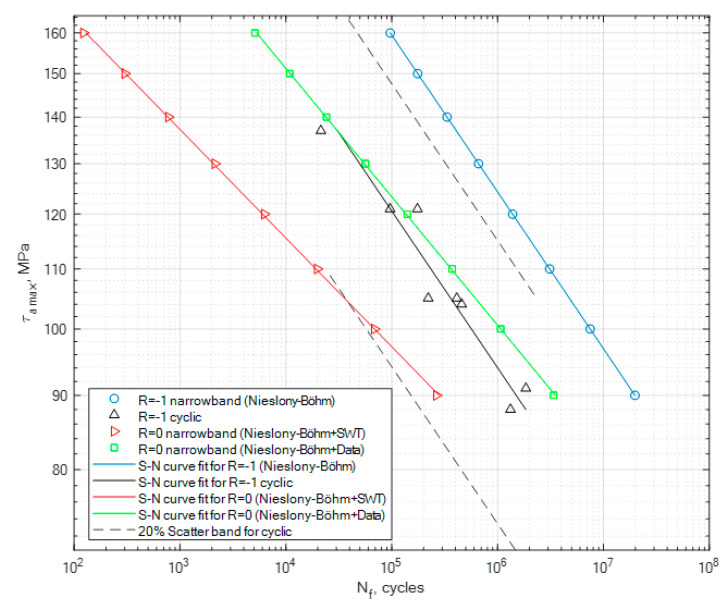
Calculation results obtained for the generated narrowband signal for the cases of *R* = −1 and *R* = 0, together with the cyclic experimental results for *R* = −1 for PA4.

**Figure 9:**
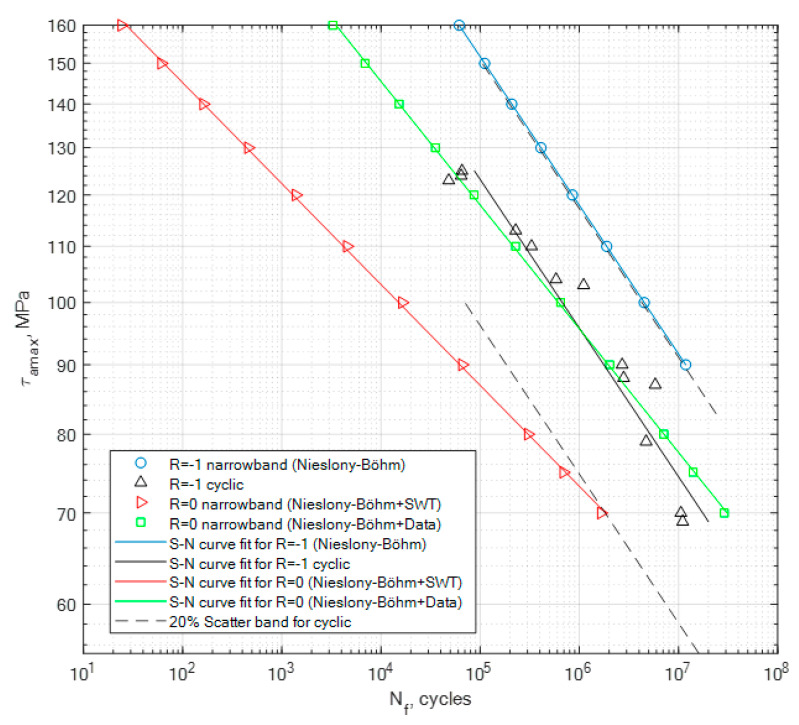
Calculation results obtained for the generated narrowband signal for the cases of *R* = −1 and *R* = 0, together with the cyclic experimental results for *R* = −1 for PA6.

**Figure 10:**
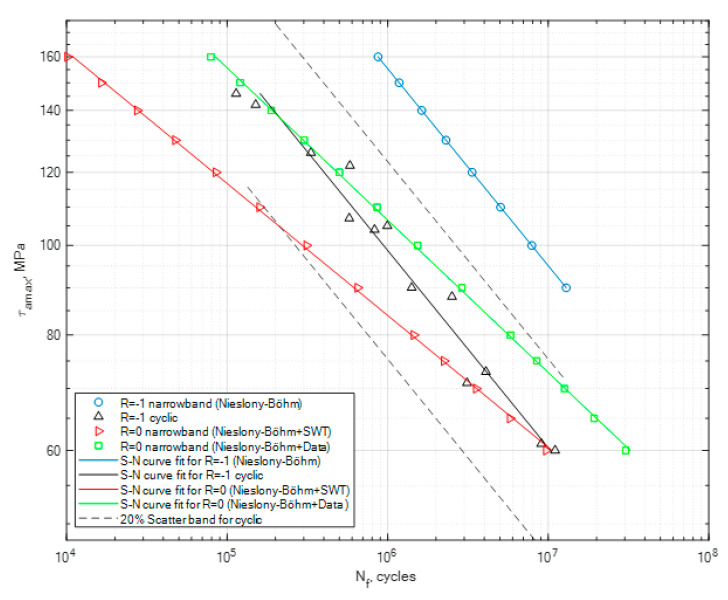
Calculation results obtained for the generated narrowband signal for the cases of *R* = −1 and *R* = 0, together with the cyclic experimental results for *R* = −1 for PA7.

The obtained coefficient values have been used in the calculation process for the generated random narrowband loading signal. This is one of the most important steps in the process of the fatigue life assessment, as in this case we want to calculate two new S-N curves for the random signal without taking the mean stress effect into account as well as taking it into account. The generated signal had a global zero mean stress value for the case of *R* = −1, and in the second case it had a global mean value equal to the global value of the maximum amplitude. In the second case, this resulted in the fact that the random generated loading signal had a value of *R* = 0. The calculation results for PA4, PA6 and PA7 for the generated narrowband signal in the cases of *R* = −1 and *R* = 0 are presented in Figure 8, Figure 9 and Figure 10, together with the constant amplitude cyclic tests. The calculations for *R* = 0 using the Niesłony−Böhm model have been presented together with the two approaches for obtaining the missing fatigue strength (SWT and literature data). The area beneath the curves represents the safe area of the generated narrowband stress signal in relation to the value of the maximum amplitude that can be applied for the given loading case.

## 5. Discussion

What seems to be important to highlight about this research is the fact that we have tested a brand new specimen shape, whose purpose was to start the initiation process mostly through shear stress. Given the shear stress distribution performed with the finite element method, we were expecting the place of the initiation of the fatigue crack to be on the surface of the specimen in the lower parts, as it was mounted to the experimental stand. The individual specimens with the non-zero global mean stress value had their crack initiation spot on the upper side of the surface due to the pre-tension of the specimen. The planned task, which was to answer the question of how this new type of diabolo sample will react to the experimental procedure seems to be clear, as it reacted exactly as we assumed. Another important point of discussion seems to be the lack of experimental results for torsional tests for these types of materials in the broader literature. Therefore, the authors hope that these results may be used by other researchers, as well as for industry applications, in order for them to perform their own tests as well as modify their calculation procedures by taking into account the mean stress effect in situations where we are missing the experimental curves for *R* = 0. The calculation procedure presented in this paper focuses on the application of two popular models, which are the Smith−Watson−Topper model for obtaining the fatigue strength value for *R* = 0 and the Niesłony−Böhm model, which serves as the transformation model for taking into account the effect of mean stress and creating reference S-N curves for the analyzed signal for *R* = −1 and *R* = 0. The second approach was to use literature data to obtain the missing fatigue strength for *R* = 0. As for the obtained results, we can note that the narrowband random results obtained for *R* = −1 are above the cyclic stress curve due to the fact that the whole stress history did not have a constant value but was a multiple level amplitudes composition. A scatter band has been generated for the constant amplitude cyclic tests with the value of 20%. As can be seen in Figure 8, Figure 9 and Figure 10, in the case of the PA6 the narrowband results for *R* = −1 are almost within this band, and in the other two cases they are way above the scatter band. Meanwhile, for the calculation results for the narrowband stress signal for *R* = 0 for the Niesłony−Böhm model + SWT, most of the results are within the 20% scatter band for PA4, PA6 and PA7. In the case where the mean stress compensation is performed with the use of the Niesłony−Böhm model + data, all results are within the 20% scatter band. Therefore, the presented method can be applied with success in order to generate S-N curves for generated random narrowband loading signals, taking into account the effect of non-zero mean stress. An important topic of discussion is the use of the SWT model in order to obtain the missing fatigue strength for the Niesłony−Böhm model. We can note that, in comparison to literature data, it underestimates the overall fatigue life, which means that it is rather a safe method. Table 8 presents the obtained calculation fatigue strengths with the use of the Smith−Watson−Topper model and the fatigue strengths obtained from the literature that have been used with the Niesłony−Böhm mean stress compensation model.

In Table 9, a comparison of the obtained ratios between the fatigue strengths for *R* = −1 and *R* = 0 obtained with the use of the two used approaches has been presented. We can note once again that the SWT model underestimates the ratio between the S-N curves.

## 6. Conclusions and Observations

The following conclusions and observations can be drawn from this paper’s results and discussion:

- The new design of the test samples has served its purpose, as it allowed us to obtain a pure shear distribution inside the sample.
- The S-N curve Niesłony−Böhm (N-B) mean stress compensation model can also be applied to torsional loading conditions.
- The literature results for the missing fatigue strength amplitude for *R* = 0 used with the Niesłony−Böhm model have improved the results substantially in comparison to fatigue strengths obtained with the SWT model.
- The calculation results obtained for the generated narrowband loading signal have allowed us to perform calculations for three new S-N curves in the case of no mean stress correction for the ratio *R* = −1, and two mean stress effect compensations for *R* = 0 with different approaches to obtain the fatigue strength for the N−B model.
- It can be noted that the narrowband curves for *R* = 0 were within the scatter band of the cyclic results for *R* = −1, within or below the 20% area, whereas the calculation results for the narrowband curves for *R* = −1 were only in the scatter band of the PA6 alloy.

## Figures and Tables

**Table 1:** Alloy description according to the different standards PA4, PA6 and PA7.

Standard				
PN	EN	WNR	ISO	DIN
PA4	AW-6082-T6	3.2315	AlSi1MgMn	AlMgSi1
PA6	AW-2017A-T4	3.1325	AlCu4MgSi(A)	AlCuMg1
PA7	AW-2024-T3	3.1354	AlCu4Mg1	AlCuMg2

**Table 2:** Important mechanical properties of PA4, PA6 and PA7.

	*R_m_* MPa	*R_e_* MPa	*E* MPa	*υ*
PA4	344 ± 3	322 ± 2	69,000	0.33
PA6	330 ± 2	312 ± 2	72,000	0.33
PA7	497	359	73,200	0.33

**Table 3:** Chemical composition of PA4, PA6 and PA7.

	Fe	Si	Zn	Ti	Mg	Mn	Cu	Cr	Other	Al
PA4	0.50	1.30	0.20	0.10	1.20	1	0.10	0.25	0.15	balanced
PA6	0.70	0.80	0.25	0.25	1	1	4.50	0.10	0.15	balanced
PA7	0.5	0.5	0.25	0.15	1.2	0.3	3.8	0.1	0.15	balanced

**Table 4:** Experimental results for the constant amplitude tests for PA4.

Specimen No.	*τ_m_*	*τ_a_*	*τ_max_*	*N_f_*
PA4-9	0	88	90	1,321,873
PA4-14	0	91	90	1,848,426
PA4-1	0	105	93	221,164
PA4-8	0	104	104	457,156
PA4-16	0	105	103	411,196
PA4-2	0	121	119	174,696
PA4-4	0	121	118	96,357
PA4-3	0	137	131	21,409
PA4-5	17	106	123	456,183
PA4-10	19	106	125	230,671
PA4-12	18	105	123	374,000
PA4-6	35	88	122	1,700,497
PA4-11	35	86	121	2,667,863
PA4-13	37	89	126	2,048,000
PA4-25	36	108	144	547,886
PA4-26	35	105	140	464,294
PA4-23	50	94	144	1,382,063
PA4-24	49	97	146	1,905,095

**Table 5:** Experimental results for the constant amplitude tests for PA6.

Specimen No.	*τ_m_*	*τ_a_*	*τ_max_*	*N_f_*
PA6-12	0	70	71	10,567,970
PA6-13	0	69	70	11,021,452
PA6-9	0	79	81	4,714,524
PA6-4	0	87	84	5,825,588
PA6-5	0	88	87	2,793,997
PA6-6	0	90	88	2,700,912
PA6-10	0	103	105	1,104,340
PA6-11	0	104	102	582,611
PA6-7	0	113	111	227,991
PA6-8	0	110	108	329,501
PA6-1	0	125	119	65,574
PA6-2	0	123	113	48,248
PA6-3	0	124	114	64,380
PA6-15	18	104	122	441,360
PA6-16	17	107	124	864,694
PA6-17	35	90	125	1,710,746
PA6-18	37	91	127	2,273,590
PA6-20	32	104	135	381,996
PA6-21	29	104	133	117,770
PA6-22	43	89	132	842,576
PA6-23	46	90	136	981,805

**Table 6:** Experimental results for the constant amplitude tests for PA7.

Specimen No.	*τ_m_*	*τ_a_*	*τ_max_*	*N_f_*
PA7-13	0	60	58	11,035,860
PA7-14	0	61	62	9,047,500
PA7-10	0	71	72	3,119,563
PA7-11	0	73	70	4,091,781
PA7-8	0	88	91	2,514,519
PA7-9	0	90	91	1,410,229
PA7-4	0	105	105	993,766
PA7-5	0	107	107	576,193
PA7-7	0	104	103	827,959
PA7-2	0	122	120	581,857
PA7-6	0	126	125	332,553
PA7-1	0	142	138	150,606
PA7-3	0	146	145	113,728
PA7-16	20	125	146	675,295
PA7-17	20	125	146	511,227
PA7-15	35	106	141	1,013,935
PA7-18	34	105	139	684,876
PA7-19	34	107	141	614,049
PA7-20	34	120	154	359,856
PA7-21	35	124	159	75,166
PA7-22	58	107	166	459,271
PA7-23	58	109	167	855,168

**Table 7:** S-N curve coefficients obtained during the torsional tests for *R* = −1.

	*m*	*τ_af_* MPa	*N_f_* Cycle
PA4	9.25	90	1,848,426
PA6	9.14	70	11,021,452
PA7	4.69	60	11,035,860

**Table 8:** Missing fatigue strength values for *R* = 0 obtained for the SWT model and from literature data.

Material	SWT	Lit. Data
	*τ_afR_* = 0 MPa	*τ_afR_* = 0 MPa
PA4	63.64	76.5
PA6	49.50	61.25
PA7	42.43	49.8

**Table 9:** Ratio between the calculated and literature fatigue strengths for the presented materials and the *R* = −1 fatigue strength.

Material	SWT	Lit. Data
	*τ*_*afR*=0_/*τ_af_*	*τ*_*afR*=0_/*τ_af_*
PA4	0.707	0.85
PA6	0.707	0.875
PA7	0.707	0.83
